# Food Sustainability Knowledge, Attitudes, and Dietary Habits among Students and Professionals of the Health Sciences

**DOI:** 10.3390/nu15092064

**Published:** 2023-04-25

**Authors:** Ainhoa Irazusta-Garmendia, Emma Orpí, Anna Bach-Faig, Carlos A. González Svatetz

**Affiliations:** 1 Faculty of Health Sciences, Universitat Oberta de Catalunya (UOC), Rambla del Poblenou 156, 08018 Barcelona, Spain; airazusta@uoc.edu (A.I.-G.); emmaorpi@uoc.edu (E.O.); cgonzalezsv@uoc.edu (C.A.G.S.); 2 FoodLab Research Group (2921 SGR 01357), Faculty of Health Sciences, Universitat Oberta de Catalunya (UOC), Rambla del Poblenou 156, 08018 Barcelona, Spain; 3 Food and Nutrition Area, Barcelona Official College of Pharmacists, 08009 Barcelona, Spain; 4 Investigador Emérito, Unidad Nutrición y Cáncer, Instituto Catalán de Oncología (ICO-IDIBELL), 08908 Barcelona, Spain

**Keywords:** sustainable diets, health science sector, eating behaviour, cross-sectional study

## Abstract

The importance of a sustainable diet is an emerging concept within sustainable food systems. Food systems emit 30% of greenhouse gases, which needs to change. A cross-sectional study was carried out to determine the knowledge, attitudes, and habits of students and professionals in the health sciences regarding a sustainable diet, comparing these to the Spanish population. We further aimed to analyse the consistency between the knowledge and attitudes of these individuals and their dietary habits and analyse the consumption of different food groups that are typical of a sustainable diet. A survey was completed by a total of 415 participants, both university students in the health sciences and health professionals. These two groups were more knowledgeable of sustainable diets than the general population, although certain concepts are unfamiliar to both populations. A positive attitude towards sustainable food habits was also observed among the population studied. The health sector reported having better eating habits than the overall population. A significant positive correlation was observed between higher fruit and vegetable consumption and deeper knowledge and more favourable attitudes. People with less knowledge and worse attitudes reported consuming more red and processed meat. The findings of this study could inform targeted interventions for health professionals given the need to promote a healthy diet but also a healthy and sustainable diet for planetary health.

## 1. Introduction

The relationship between food and health has been studied for many years [1]. However, the connection between what humans eat and the ecosystem was not studied until the mid-19th century [2]. The most recent report of the Intergovernmental Panel on Climate Change shows that global temperatures will continue to rise until at least the middle of the century if we do not change our production and consumption practices [3,4]. The report further indicates that unless drastic reductions are made to CO_2_ and other greenhouse gas (GHG) levels in the coming decades, global temperatures will rise by 1.5 to 2 °C or as much as 2.7 °C during the 21st century [5].

It has become clear that this climate change is the result of human activity, and current rates of consumption and production will lead to worsening access to education, heightened inequality, less access to food and drinking water, and greater hunger among the world’s population. For this reason, world leaders at the United Nations developed the Sustainable Development Goals (SDGs) to eradicate poverty, protect the planet, and ensure prosperity for everyone [6].

The food system is responsible for more than one-third of global GHG emissions, accounting for 28–34% of emissions overall [7]. Not only must we take into account what is consumed, as food waste is also an important factor. It is estimated that one-third of all food produced each year is wasted in consumer and retail settings [8].

There is sufficient evidence demonstrating that the consumption of meat and animal products, in general, are the most important generators of GHGs in the food system, which is detrimental to the health of the population [9]. On the contrary, plant-based foods (e.g., fruits, vegetables, grains, legumes) are not only healthy but generate much lower GHG emissions [10].

In recent years, the Spanish population has moved away from the Mediterranean diet towards a more Western consumption pattern, which is known to be associated with numerous chronic diseases and a much greater environmental impact [11,12,13,14]. This is a matter of concern for both human health and the environment, jointly referred to as planetary health.

According to the EAT-Lancet Commission [15], continuing to follow this dietary pattern will worsen the burden of non-communicable illnesses such as cardiovascular disease, cancer, and obesity, as well as communicable diseases including COVID-19 [16]. Moreover, it will increase the negative impact of food production on GHG emissions, nitrogen and phosphorus pollution, biodiversity loss, and inefficient use of water and land, thus reducing the stability of the earth’s system. The recommendations of the report included a minimum 50% reduction in the overall consumption of unhealthy foods, such as red meat and sugar, and a more than 100% increase in the consumption of healthy foods, such as fruits, vegetables, and pulses [15].

Most studies of knowledge and attitudes regarding sustainable diets support the need for initiatives aimed at educating consumers about sustainable dietary patterns [17,18]. A study in a representative sample of the Spanish population [19] revealed an interest in sustainable food, although only 40% of the population rated sustainable diets as healthy. However, knowledge on the subject remains cursory. The study reported that more than 50% of the population was unaware of the environmental impact of meat, and this figure increased to 70% regarding the impact of fish and dairy products.

As misconceptions persist amongst the general public, informational and educational interventions seem necessary. Goal 13 of the SDGs focuses on the need for climate action and urgent changes to mitigate the adverse effects of climate change and prevent avoidable alterations to the ecosystem for the time being [6].

Despite extensive research on the need to adopt sustainable diets, there is limited research on what knowledge and attitudes are towards sustainable diets in the health sector. The role of healthcare professionals is fundamental in promoting health, and a healthy diet is essential to prevent diet-related risk factors, such as overweight and obesity and associated noncommunicable diseases [20,21]. Furthermore, it is essential for healthcare professionals to have deeper knowledge of sustainable diets and to act in accordance with their knowledge and attitudes, since they are responsible for disseminating this information to patients and the general population. Furthermore, higher education has great potential to aid in achieving the SDGs and to formally and informally transfer knowledge of food sustainability to students.

For this reason, individuals responsible for disseminating this information must be aware of the importance of including food sustainability in their efforts and be able to implement these initiatives in communities. While research has been carried out in the general population, no studies on the topic have been based on a sample of both healthcare professionals and students. Knowledge and perceptions of food and environmental sustainability in university populations have been previously explored [22,23] in Spain, although with greater focus on environmental issues, SDGs, and related concepts. Research assessing the knowledge of actual food habits and attitudes toward dietary customs is more scarce.

Therefore, before designing strategies and proposing to include them in the educational curriculum, it seems necessary to survey existing knowledge, attitudes, and habits surrounding sustainable food. Therefore, this study evaluated the knowledge, attitudes, and eating habits of students and professionals, who are current or aspiring members of the healthcare system, and compared them to those of the Spanish population. The findings of this study will enable us to assess possible gaps in the knowledge, attitudes, and eating habits within the health sector in order to inform targeted interventions concerning this important topic given the need to go beyond promoting just a healthy diet but also a healthy and sustainable diet for the well-being of the planet.

## 2. Materials and Methods

### 2.1. Sample and Study Design

A cross-sectional study was conducted by administering online questionnaires to students and professionals of the health sciences between late December 2021 and April 2022, based on participant availability. A convenience sample of participants was divided into two large samples: the first comprised two subsamples of university students, and the second was made up of health professionals consisting of *Universitat Oberta de Catalunya* (UOC) health sciences faculty professors and members of the Barcelona Official College of Pharmacists. Professionals from a variety of fields within the health sciences were recruited via e-mail, such as members of the Official College of Pharmacists.

Questionnaires were also administered to nursing degree students of the Donostia/San Sebastian and Leioa campuses belonging to the Faculty of Medicine and Nursing of the University of the Basque Country/*Euskal Herriko Unibertsitatea* (UPV/EHU). In addition, university school of nursing students of Vitoria-Gasteiz, which is affiliated with the UPV/EHU, were also included. In total, 260 responses were collected from 1300 students, which represents a 20% response rate. Parallel to this, questionnaires were administered to UOC students enrolled in different academic programmes within the health sciences, including undergraduate and master’s degrees, other postgraduate studies and specialisation programmes, and doctoral studies. These questionnaires were answered by 155 people. In total, 415 individuals took part, including university students and health professionals.

The survey contained an informed consent form indicating that participation in the survey was completely voluntary and that the responses would be anonymous. The study was conducted in compliance with the ethical principles for research involving human beings and the processing of personal data as set forth in the Declaration of Helsinki and was approved by the Ethics Committee of the Open University of Catalonia, CE22-PR03.

### 2.2. Research Tools

To obtain data on knowledge and attitudes, we adapted a validated questionnaire from a study carried out in the Spanish population [19] to the characteristics of our population. Thus, the original survey (Table S1 (Supplementary Material)) was adapted from a study of the general Spanish population [19] for use in the health sector. We included only three additional variables regarding food waste to the original survey of the general population. The tool was pre-validated in a small panel of experts to assess the relevance and appropriateness of the questions. Additionally, the process of administering the survey was then piloted on a small sample of people known to the authors to verify the comprehensibility and answerability of the items. The questionnaire included the following:

1. Participant data.
2. Knowledge of concepts related to climate change. Possible answers were Yes/No/Unknown (DK).
3. Priority assigned to a list of sustainable food concepts. Participants completed this item using a Likert scale with values ranging from 0 to 5, with 0 indicating DK/No response (NR), 1 not at all important, and 5 very important.
4. Impact of different types of foods on the sustainability of the planet. Possible answers were DK/High impact/Medium impact/Low impact.
5. Importance of water in the production of plant and animal products. Possible answers were plant-based products, animal-based products, and DK.
6. Attitude (three questions) towards a sustainable diet (Supplementary Materials Table S1). Responses were given on a Likert scale ranging from 1 to 5, with 1 indicating not at all important and 5, very important.
7. Food waste (three questions, Supplementary Materials Table S1). Possible answers were as follows: no consumption, never, rarely, sometimes, often, and always.
8. Food consumption frequency, using a validated questionnaire [24]. Possible answers were as follows: Never or almost never/1–2 times a month/1–2 times a week/3–5 times a week/1–2 a day/>3 a day.

Knowledge was correlated with attitudes and eating habits to analyse the consistency between the two. Answers given in Items 2 to 5 were used to design an index of sustainable food knowledge. To obtain a score that could be used for comparisons with attitudes scores, the answers were coded as indicated.

When coding Item 3 (Priority assigned to a list of sustainable food concepts), the answers very important and important were assigned a score of 3, moderately important a score of 2, unimportant or not important a score of 1, and unknown a score of 0. The options no additives, minimally processed products, few ingredients and easy to follow were discarded because they were the least relevant in terms of sustainable food.

When coding responses for Item 4 (Impact of different types of food on the sustainability of the planet), the scores assigned were 0, DK; 1, High impact for foods with a low environmental impact (plant-based foods) and low impact for foods with higher impact (processed meat, red meat, and dairy); 2, Medium impact for any type of food; and 3, Low impact for foods with a low environmental impact (plant-based foods) and high impact for foods with higher impact (processed meat, red meat, and dairy). The higher the score, the greater the knowledge.

For the attitude index (Items 6 and 7), the answers from Item 6 (Attitude towards a sustainable diet) were linked into three options, following the same pattern used in the sections on knowledge. The cognitive, affective, and potential behavioural dimensions of attitude towards a more sustainable diet were evaluated.

For coding of Item 7 (Food waste), the answers never and rarely were linked and scored, with 3 being the highest score; answers of sometimes were scored with a 2, and more frequently was given a score of 1. Therefore, participants reporting the least waste obtained the highest scores.

For Item 8 (Food consumption frequency), the foods selected were red meat, processed meat, plant-based food, and dairy, as these are the most important in terms of sustainability. Answers were coded to obtain weekly consumption frequencies: never or almost never was assigned a weekly frequency of 0; 1 to 2 times a month was given a weekly frequency of 0.375; 1 to 2 times a week a frequency of 1.5; 3 to 5 times a week a frequency of 4; 1 to 2 times a day a frequency of 10.5; and more than 3 times a day a weekly frequency of 21.

### 2.3. Statistical Analyses

Results for categorical variables (e.g., knowledge) were expressed as frequencies and percentages. Data obtained for continuous variables were expressed as mean ± standard deviation. The normal distribution of quantitative variables was analysed using the Kolmogorov–Smirnov test. Statistical differences between percentages among the different socio-demographic groups were analysed by chi square test. Spearman correlations were performed between the consumption frequencies with the score of the sum of knowledge and attitudes. The SPSS programme was used for statistical analysis. Differences were considered statistically significant when *p* < 0.05.

## 3. Results

### 3.1. Characteristics of Participants

Table 1 shows the characteristics of the study participants. Four hundred fifteen individuals completed the questionnaire. Among them, 361 were women, 52 men, and 2 identified as nonbinary. Of the overall study population, 78.8% were students of the health sciences and 21.2% were health professionals.

We assessed whether there were statistical differences between socio-demographic groups, comparing the percentages of responses to questionnaire items; no relevant differences were observed. Given the lack of differences and the relatively small number of professionals, we decided to perform a joint analysis.

The mean age was 27.73 years (range, 18–76 years). Data were gathered on the size of the town or city where the participants resided as well as their monthly household income.

### 3.2. Knowledge of Concepts Related to Sustainability

The term “environmental footprint” was familiar to 56.10% of subjects studied. Sixty percent of participants had knowledge of the concept of carbon footprint, and the term “biodiversity” was known by 89.80% of the sample. Greenhouse gas emissions (GHG) was the most widely known concept (92.00%). In contrast, the term “water footprint” was the most scarcely known concept (12.30% of participants).

### 3.3. Priority Assigned to Different Concepts Related to a Sustainable Diet

As shown in Table 2, respondents indicated the following qualities as very important in sustainable food: being healthy for humans (70.36%), consisting of fresh products (62.65%), produced locally (62.41%), respectful of biodiversity (61.69%), and having a low environmental impact (60.24%). In contrast, the lowest score was given for presence of few ingredients, as only 14.70% of the participants believed this criterion to be very important.

In the following question, participants were asked to indicate the environmental impact they believed certain foods had (Table 3). Most participants reported that plant-based foods have a low environmental impact (53.01%). In contrast, a wide majority declared that ultraprocessed foods (84.58%), processed meats (82.89%), and red meat (75.42%) had a high impact. Dairy products were reported to have a moderate (44.82%) or high (40.00%) environmental impact.

### 3.4. Participant Attitudes towards Sustainable Food

Participants were asked three questions on how willing they are to consume sustainable food and how important sustainable food is to them.

Regarding sustainable food production, 28.91% of respondents considered this very important, 41.69% important, 25.06% somewhat important, and 4.34% not very or not at all important.

For the second question, which asked whether they would be willing to pay more money for food and beverages that are produced more sustainably, 12.53% indicated that they would be very willing, 46.5% willing, 31.09% somewhat willing, and 9.88% stated that they would be somewhat or not at all willing to pay more money for sustainable food.

Finally, participants were asked whether it is important for them to buy sustainable food: 44.82% stated that it is important, 19.52% very important, 28.19% somewhat important, and 7.47% not very important or not important at all.

### 3.5. Habits Regarding Food Waste

With regard to waste, respondents answered a range of questions, such as how often they usually leave food on their plate or throw away spoiled food from the refrigerator or pantry. Regarding the former, 84.34% of the participants answered that they never or rarely (44.82% and 39.52%, respectively) leave food on their plate. Only 10.12% responded sometimes, and 5.3% often.

As for disposing of spoiled food from the refrigerator, the results were similar, although 16.63% responded “never” and 51.32% “rarely”. “Sometimes” was chosen by 21.93% of participants. “Often” was the answer given by 8.67% and “Always” by 1.45%.

### 3.6. Frequency of Food Consumption Habits in Terms of Sustainable Eating

Overall, fruit and vegetables were consumed 1–2 or more times a day by 67.47% and 62.89% of the participants, respectively. Dairy products were also consumed 1–2 times a day by 48.19% of the sample. Red meat, on the other hand, was consumed 1–2 or fewer times a week by 82.89% of the respondents. Finally, the greatest discrepancy in terms of frequency of consumption was observed for processed meat, as 32.29% reported that they never eat processed meat, with another 20% eating it 3–5 times a week (Table 4).

### 3.7. Relationship between Sustainable Food Knowledge and Attitudes and Eating Habits

When we correlated data on knowledge and attitudes concerning sustainable food with the frequency with which participants consumed different food groups having a more widely recognised impact on the environment, significant associations were seen. A significant positive correlation was found between deeper knowledge and positive attitudes regarding sustainable food and higher consumption of fruit and vegetables. In contrast, consumption of red and processed meat was negatively associated with knowledge and positive attitudes toward sustainability, reaching statistical significance. Regarding dairy, a negative correlation was observed, but the association was not significant (Table 5).

## 4. Discussion

A number of studies on sustainable food have been carried out in Spain [19,22,23]. To the best of our knowledge, ours is the first to evaluate attitudes, knowledge, and habits regarding sustainable foods among students and professionals of the health sciences.

Nearly all participants (90%) had an accurate idea of what a healthy diet is, stating that it is “very important” or “important” for a healthy diet to be healthy for humans, based on plant-derived products, accessible, and include fresh and locally sourced products (Table 1). Almost 90% of the participants answered that sustainable food has to be “zero waste”. Interestingly, over 70% stated that a healthy diet should be free of additives, and 57% of the participants believed that it should be minimally processed.

In the study performed in the Spanish general population [19], conducted via phone-based survey, 2052 valid questionnaires were included. The main study objective was to evaluate the knowledge of food sustainability and environmental impact as concepts in a representative sample of the Spanish adult population, in addition to assessing related attitudes and behaviours. The statements given the highest response rates by respondents were “respectful of biodiversity” and “abundant in fresh fruits”, but they did not include “healthy for humans”. These differences may be indicative of a greater priority assigned to health by students of the health sciences and health professionals than the general Spanish population.

The UB survey [23] draws on data from 1220 participants, including teaching staff and students of the health sciences. Survey participants indicated that the concepts “high waste”, “hygiene”, “plastic packaging”, and “fat content” were of greater importance. The authors concluded that the survey participants had moderate/low knowledge of sustainability, as only 10% fully identified a healthy diet with sustainability. This may be due to the fact that nearly half of the participants were between 51 and 65 years of age, and only 18% were students.

As for the question on the environmental impact of different foods (Table 2), only 12.3% of respondents stated that plant-based foods have a high environmental impact. More than 90% of the participants believed that red and processed meat and ultraprocessed foods have a high environmental impact. However, findings from the study conducted in the general Spanish population reveal an absence of this knowledge, since more than 50% of the sample answered that meat and its derivatives have a positive impact on food sustainability [19].

Positive attitudes toward sustainable food were found in both the general Spanish population and the participants of the present study. This indicates that sustainable food is a topic of social interest, and that deeper knowledge of this issue may lead the population to change their habits.

Sixty percent of the individuals surveyed in our study consider it “very important” or “important” for food to be produced sustainably. Furthermore, over half stated that they are willing to pay more to consume a sustainably produced diet. In the other study [23], 59% acknowledged that they take sustainability into account when shopping.

Similarly, among university students [23] it was detected that women have a more favourable attitude towards sustainable food and are more likely to purchase and consume organic food than men. However, it seems that men are willing to pay more for more this type of food, although this cannot be shown in our study due to the relatively small percentage of male participants.

Our results show that reported food waste is quite low and that fruits and bread appear to be the most commonly wasted items. Another study of university students [25] found a similar percentage of food waste. In university students [23], participants recognised that they waste food occasionally. These results may contradict existing data since, according to the UN [26], 121 kg of food annually are wasted per capita around the world, and 74 kg (roughly 200 g per day) are wasted in households.

One of the reasons for this difference may be that students often do not take responsibility for cooking in their homes and do not realise the waste that is created. Additionally, they are most often not the ones who shop, plan weekly meals, or dispose of expired food.

The habits of our study participants (Table 3) show a lower consumption of red and processed meat compared to the general population and a higher consumption of vegetables [27]. Almost two-thirds consume fruits and vegetables daily. This may be due to a more acute environmental awareness, but it may also result from a deeper knowledge and awareness of recommendations to decrease red meat consumption and increase intake of plant-based foods due to health concerns. It should also be taken into account that these respondents were mostly women, who tend to be more health-conscious and eat less meat [28] and more vegetables [29] than men [28].

Milk and dairy products are consumed daily (58%) by the participants. However, current recommendations on dairy intake are inconsistent. On the one hand, health professionals in recent years have recommended limiting consumption of dairy products or consuming low-fat dairy foods due to their association with health problems, although scientific evidence on the topic [30] seems to challenge this recommendation of low-fat dairy for health reasons; dietary guidelines have changed in this regard, and greater emphasis is now given to reducing added sugars [31]. Even so, the most recent recommendations, such as the Harvard Plate [32], recommend limiting consumption to a maximum of 1–2 servings per day.

Furthermore, it has been shown that dairy products have a high environmental impact due to their close link to livestock farming. Therefore, in terms of sustainability, it is recommended to limit the consumption of dairy products [33].

According to our results, those participants who had more knowledge and more favourable attitudes towards sustainable food ate significantly more fruits and vegetables and less red and processed meat (Table 4). Conversely, respondents with less knowledge and worse attitudes to food sustainability consumed significantly more processed and red meat. It has been shown that education increases environmental concern and also influences behaviour, especially in women [34]. Therefore, it is to be expected for nursing students to have better habits than the general population.

The effects of climate change and a move toward sustainable eating habits have been on the rise in recent years and are becoming increasingly important, especially on social media. This has led to improvements in the eating habits of young people [35]. In another study [36], which was carried out in 2015 in the Basque Country, the consumption of different food groups among students of the health sciences (most of them nursing students) was measured. The results showed that both women and men consumed more lean and fatty meats as well as sweets and pastries and showed a lower consumption of grains, fruits, vegetables, olive oil, fish, nuts, and legumes than in the present study. Therefore, it can be said that the eating habits of nursing students may have improved since the research in 2015.

Socioeconomic status is also related to healthy habits and health literacy [36], so it is unsurprising that the population group analysed in this study reported better habits than the general population, since people with lower socioeconomic levels cannot afford healthier and more nutritious foods.

Based on the results presented above, it is clear that health education on sustainable foods is important. In addition, it has been shown that health education and promotion interventions can be effective [37].

To this end, the role of nurses and health professionals is essential because they are involved in disseminating knowledge of healthy habits throughout the population and encouraging people to put them into practice [38].

However, when changes are based on something so integrated into the culture and society as food, it is often difficult to make these lifestyle adaptations [39]. Regarding changes in eating habits for environmental reasons, reducing meat consumption is the least accepted measure according to the population, since meat is a staple in our society, and people find it difficult to reduce or eliminate it altogether [40,41].

Changes to diet may require challenging socio-cultural norms and practices and making sustainably produced food more available and affordable. Toward this end, policymakers responsible for fostering a sustainable and healthy diet should take the appropriate measures [20].

However, given the scientific evidence [20] in Spain that unhealthy foods are cheaper than healthy alternatives, and that price is one of the most important factors in consumer decision-making, several strategies must be implemented simultaneously in accordance with recommendations of the WHO [42] regarding fiscal measures. This means reducing the cost of healthy foods for consumers and providing food incentives while simultaneously introducing taxes to reduce consumption of unhealthy and unsustainable foods. In a recent study of the ecological impact of food habits among the Spanish population [43] based on data from a representative sample of the general population, it was found that, for 65% of the population, the biggest obstacle to changing habits was the price of food with a low environmental impact. On the other hand, when asked which measures would help them the most, 53.4% answered that reducing the price of foods with a lower impact would help them considerably in changing their habits and 33.1% believed this would help considerably. In contrast, there is no general consensus on the issue of increasing prices of foods with a high environmental impact, as 31.6% of the respondents stated that such measures would help them considerably and 27.8% even more so. Raising prices was found to be more popular among young, university-educated, and rural people, and less popular among those who are less educated and live in large cities [44].

Several limitations should be highlighted despite the novelty of the investigation in the target population, particularly for the Spanish context. Firstly, the small sample size and underrepresentation of health professionals in comparison to students are amongst the main limitations of the study, along with the use of convenience sampling, which may have led to participation bias. Furthermore, the sample was limited to certain sectors within the health sciences (e.g., medicine, nursing, pharmacy, psychology, nutrition), but is not representative of all fields. Another aspect to consider is that a majority of participants were women, which may be a result of the increasingly disproportionate representation of women in the health sector and the fact that women tend to be more willing to participate in surveys. Consequently, the transferability of these findings to health professionals in other contexts should be considered with caution. Secondly, possible biases in self-reported dietary habits inherent to questionnaires on frequency of intake may have influenced our findings. Thirdly, in addition to the pre-validation of the survey tool performed, an assessment of the validity and reliability of the modified survey would have been optimal. Although further research in larger and more representative samples of the health sector is needed for purposes of generalizability, the study is relevant, since the health sector could contribute to transforming food demand toward practices that are healthier and more sustainable.

## 5. Conclusions

According to our results, health science students and professionals, as expected, have greater knowledge of sustainability than the general Spanish population. However, attitudes towards sustainable diets are positive in both the general population and the students and professionals surveyed. Individuals belonging to the health sector have better eating habits than the general population in terms of both healthiness and food sustainability. Health science students and professionals, with greater knowledge and more favourable attitudes towards sustainable diets, have better eating habits in terms of consuming more fruits and vegetables and less red meat. The findings of this study could inform targeted interventions in health professionals given the need for healthcare workers to go beyond promoting a healthy diet and also advocate a healthy and sustainable diet for the health of the planet.

## Figures and Tables

**Table 1:** Characteristics of the study participants (*n* = 415).

	*n*	%
Gender (*n*)		
- Female	361	86.99%
- Male	52	12.53%
- Nonbinary	2	0.48%
- Total	415	100%
Age		
- Mean age	27.73	
- Standard derivation	11.87	
Occupation		
- Students	327	78.80%
- Healthcare professionals	88	21.20%
- Total	415	100%
Population		
- >10,000	288	69.29%
- 5000–10,000	71	17.10%
- <5000	56	13.40%
- Total	415	100%
Monthly household income		
- >4001 euros	73	17.60%
- 3001–4000 euros	95	22.89%
- 2001–3000 euros	142	34.22%
- 1001–2000 euros	90	21.68%
- <1000 euros	15	3.66%
- Total	415	100%

**Table 2:** Opinion on the relative importance of the components of a sustainable diet (%).

	Not Important at All		Less Important		Somewhat Important		Important		Very Important		Do Not Know		Total
Reduced impact		(0.00%)	1	(0.24%)	41	(9.88%)	116	(27.95%)	250	(60.24%)	7	(1.69%)	415 (100%)
Biodiversity		(0.00%)	2	(0.48%)	26	(6.27%)	126	(30.36%)	256	(61.69%)	5	(1.20%)	415 (100%)
Additive-free	3	(0.72%)	26	(6.27%)	69	(16.63%)	135	(32.53%)	174	(41.93%)	8	(1.93%)	415 (100%)
Minimally processed	1	(0.24%)	12	(2.89%)	47	(11.33%)	121	(29.16%)	206	(49.64%)	28	(6.75%)	415 (100%)
Few ingredients	36	(8.67%)	114	(27.47%)	101	(24.34%)	87	(20.96%)	61	(14.70%)	16	(3.86%)	415 (100%)
Organic products	2	(0.48%)	18	(4.34%)	51	(12.29%)	153	(36.87%)	184	(44.34%)	7	(1.69%)	415 (100%)
Fresh products	1	(0.24%)	4	(0.96%)	23	(5.54%)	119	(28.67%)	260	(62.65%)	8	(1.93%)	415 (100%)
Plant-based	1	(0.24%)	1	(0.24%)	29	(6.99%)	125	(30.12%)	249	(60.00%)	10	(2.41%)	415 (100%)
Local products	4	(0.96%)	5	(1.20%)	29	(6.99%)	110	(26.51%)	259	(62.41%)	8	(1.93%)	415 (100%)
Accessible		(0.00%)	7	(1.69%)	32	(7.71%)	131	(31.57%)	235	(56.63%)	10	(2.41%)	415 (100%)
Easy to follow	2	(0.48%)	16	(3.86%)	61	(14.70%)	140	(33.73%)	181	(43.61%)	15	(3.61%)	415 (100%)
Healthy for humans		(0.00%)	4	(0.96%)	19	(4.58%)	89	(21.45%)	292	(70.36%)	11	(2.65%)	415 (100%)
Zero waste		(0.00%)	3	(0.72%)	32	(7.71%)	124	(29.88%)	241	(58.07%)	15	(3.61%)	415 (100%)

**Table 3:** Opinion on the environmental impact of the main food groups (%).

	High Impact		Moderate Impact		Low Impact		Do Not Know			Total
Plant-based	51	(12.29%)	123	(29.64%)	220	(53.01%)	21	(5.06%)	415	(100%)
Red meat	313	(75.42%)	78	(18.80%)	12	(2.89%)	12	(2.89%)	415	(100%)
Processed meat	344	(82.89%)	47	(11.33%)	12	(2.89%)	12	(2.89%)	415	(100%)
Ultraprocessed food	351	(84.58%)	37	(8.92%)	9	(2.17%)	18	(4.34%)	415	(100%)
Milk	166	(40.00%)	186	(44.82%)	38	(9.16%)	25	(6.02%)	415	(100%)

**Table 4:** Frequency of consumption of different food groups (%).

	Never		1 Time a Month		1–2 Times a Week		3–5 Times a Week		1–2 Times a Day		>3 Times a Day			Total
Fruits	8	(1.93%)	6	(1.45%)	42	(10.12%)	79	(19.04%)	200	(48.19%)	80	(19.28%)	415	(100%)
Vegetables	1	(0.24%)	1	(0.24%)	45	(10.84%)	107	(25.78%)	207	(49.88%)	54	(13.01%)	415	(100%)
Dairy	28	(6.75%)	19	(4.58%)	42	(10.12%)	85	(20.48%)	200	(48.19%)	41	(9.88%)	415	(100%)
Red meat	86	(20.72%)	81	(19.52%)	177	(42.65%)	69	(16.63%)	2	(0.48%)	0	(0.00%)	415	(100%)
Processed meat	134	(32.29%)	95	(22.89%)	53	(12.77%)	83	(20.00%)	50	(12.05%)	0	(0.00%)	415	(100%)

**Table 5:** Correlation between knowledge and attitudes about sustainable eating and eating habits.

Food Groups	Spearman’s Rho Correlation *	Significance, *p*-Value
Vegetables	0.303	<0.001
Fruits	0.161	<0.001
Dairy	−0.062	0.205
Red meat	−0.230	<0.001
Processed meat	−0.158	0.001

* Rho: Spearman’s coefficient of correlation between knowledge and attitudes on sustainable eating and eating habits.

## Data Availability

The datasets generated during and/or analyzed during the current study are not publicly available due to privacy or ethical restriction but are available from the corresponding author upon reasonable request.

## Supplementary Materials

The following supporting information can be downloaded at: https://www.mdpi.com/article/10.3390/nu15092064/s1, Table S1: Survey of knowledge, attitudes, and habits regarding sustainable food.
