# Supplementary material for: Food Sustainability Knowledge, Attitudes, and Dietary Habits among Students and Professionals of the Health Sciences
Source: Nutrients. 2023 Apr 25;15(9):2064. doi: 10.3390/nu15092064 (PMC10180935; doi:10.3390/nu15092064)
Supplement: Supplementary file 1 [file nutrients-15-02064-s001.zip › nutrients-2338966-supplementary.pdf]

Supplementary Materials:

Table S1. Survey of knowledge, attitudes, and habits regarding sustainable food.

An informed consent statement explaining what the study consisted of appeared before the questions.

- **Block 1: Participant data**

Gender, age, student/worker, profession, academic year, population of the place of residence of the participant, family monthly income (euros), and the type of family diet in recent months.

- **Block 2: Are you familiar with the following concepts?**

Environmental footprint, carbon footprint, biodiversity, greenhouse gases, water footprint. Possible answers were Yes/No/DK

- **Block 3: To what extent do you consider that each of the following aspects contribute to sustainable food?**

Reduced environmental impact, respectful for biodiversity, no additives, minimally processed products, few ingredients, organic products, many fresh products, diet rich in plant-based foods, locally produced products, culturally typical product, accessible, easy to follow, healthy for humans, with little waste. Answers were provided according to a Likert scale, with scores ranging from 0 to 5: 0 indicated DK/NC; 1, not important at all; and 5, very important.

- **Block 4: Indicate the impact that you think the following foods have on the sustainability of the planet**

Plant-based food, red meat, processed meat, white meat, fish and seafood, processed foods, oils, nuts, milk and dairy products, eggs, soft drinks, and processed beverages. Possible responses were DK/ High impact / Moderate impact/ Low impact. Which product do you think needs more water for consumption? Responses were plant-based products, animal products, or DK.

- **Block 5: Attitude towards a sustainable diet**

How important is it for you that the products you consume be sustainably produced?; To what extent would you be willing to pay more money for sustainably produced food and

beverages?; How important is it for you to buy sustainable food?
Responses were given according to a Likert scale from 1 to 5, with 1 being not important at all, and 5 being very important.

- **Block 6: Food waste**

-State how often you leave food on your plate.

-State how often you throw away spoiled food from the refrigerator or pantry.

-State how often you waste the following foods: legumes, fish, pasta, meat, eggs, milk, yogurt, cheese, vegetables, fruit, bread, and sweet or savoury snacks.

The following options were available with regard to waste: no consumption, never, rarely, sometimes, often, and always.

- **Block 7. Food consumption frequency (22)**

State how often you consume the following foods: legumes, fish, pasta, meat, eggs, milk, yogurt, cheese, vegetables, fruit, bread, sweet or savoury snacks, alcoholic beverages, sweetened soft drinks, low-calorie soft drinks.

Responses in terms of consumption frequency were given in accordance with the following options: never or almost never/ 1-2 times a month/ 1-2 times a week/ 3-5 times a week/ 1-2 a day/ >3 a day.

The questionnaire is based on closed-response questions (between 3 and 6 Likert-type items).
